# Is War Dysgenic?

**Published:** 1915-08

**Authors:** 

**Affiliations:** San Antonio


### The Department of Eugenics, Mental and Social Hygiene

CONDUCTED BY
DR. MALONE DUGGAN
813-814 Gibbs Building, San Antonio, Texas
All communications for this department should be sent to DR. DUGGAN

### Is War Dysgenic?

Is war dysgenic? That is to say, does war make against eugenics? Or, in other words, what will be the effect of a war, such as the present European carnage, on future generations? If it will not improve the heritable character of the next generation it will be dysgenic. Mere internecine strife, unwanton destruction of property, however, does not in itself necessarily mean the destruction of characters suitable for inheritance. There are many things more destructive to the race than war. Slavery, social degeneracy and dishonor of the State may be worse than war and result in far greater dysgenic effects. It is wholly conceivable that war may prove helpful to a country already top-heavy with over-civilization, if by unusual sacrifice the people are compelled to resort to the necessities of life, provided the finer germplasms are not all stamped out. War may awaken a stronger national consciousness and idealism; it may arouse the dormant fires of heroism and martial virtues; it may stimulate the virtue of personal sacrifice and hardihood; it may even be eugenic in its effects, by demanding the civic duty of parenthood and the safeguarding the race from racial deteriorations. Nothing is more wholesome, sometimes, than deep remorse and penitence that will awaken a consciousness to personal responsibility and the rights of others.

But, what is the price of war? Admitting that the above may be advantages gained, what is the cost of our natural and social heritage? Biologically, war is wasteful since it prunes off a disproportionately large number of people which society can least afford to lose. It substitutes natural selection for rational selection, thus reverting back to the crudest primitive period when war was a factor in weeding out the unfit. It lowers physical vigor among the non-combatants, which results in the arrest of development. As Prof. J. Arthur Thompson says, "While the germplasm is not specifically affected, yet it is quite conceivable that very unfavorable natural conditions may induce prejudicial germinal variations of a heritable sort."

In order to better understand the relation of heredity in the evolution of the race we should have a clear idea of what is meant by survival of the fittest. If man followed natural selection, as does the vegetable kingdom, the fittest would not survive. In his development there is a far more important consideration. Social heredity, perhaps, more than natural heredity determines the result; which is to say, it is not only the individual but his environment as well that must be considered. There must be the subordination of the individual to the species. A part of the individual's fitness to survive is his being capable of self-sacrifice. There can be no evolution without discrimination in the selection of characters worthy of survival. Masses count for naught except in serving the finer characters in society. The effect of war is to (by the destruction of everything but the bare necessities) so deprive these finer spirits of their necessary stimuli that they will perish. Specialism is the result of hundreds of years of accumulating social progress and cannot be easily replaced. Indeed, greater progress could be made, building over the ruins of a devastating war, from the remaining social characters than from inheritance. In this light, war, in destroying our social heritage, as well as lowering the physical standard, leaving out of consideration the suffering and mental anguish entailed, is dysgenic.

From out of the gloom there is the hope, however, as expressed by Prof. Thompson: "In the eugenic prospect of developing a great confederate organization for the common task of lasting peace. A possible source," as he states, "of variational stimuli regenerating even the permplasm."—*Eugenics and War: Popular Science Monthly*, May, 1915.

